# Book Reviews

**Published:** 1833-03

**Authors:** 


### BIBLIOGRAPHICAL NOTICES.

*A Demonstration of the Nerves of the Human Body.* Parts II. and III. *Containing Descriptions and Illustrations of the Lumbar and Sacral Portions of the Sympathetic and the Nerves of the Abdominal Viscera and the Cerebral Nerves.* By JOSEPH SWAN.—Imperial folio. London: Longman and Co.

THIS is, perhaps, the most splendid anatomical work ever presented to the profession in this country; and, in point of utility also, it may fairly compete with any other publication of the kind. It certainly is very desirable that every anatomical student should make himself master of every part of the structure of the human body by his own manual dissection; but, like many other objects that are much to be wished for, this is one that can rarely be obtained. Very few students would have the patience, and very few the requisite dexterity in the use of the scalpel, to trace out the innumerable branches and connexions of many of the nerves, and yet it is absolutely necessary that this information should be obtained; for, without it, the medical or surgical practitioner must constantly be involved in doubt and perplexity: he must be utterly unable to account for the origin, or even to suspect the nature, of many curious, and even extraordinary, morbid phenomena which so frequently arise in the course of nervous diseases. How unintelligible, for instance, how perfectly bewildering, must often be the symptoms which occur from spinal irritation in hysterical complaints, to the practitioner who does not know the various and complicated connexions of the spinal and great sympathetic nerves, and the many important organs they supply with nervous influence, and which must consequently sympathize with, and suffer from, any lesion affecting them. It may be said that all the knowledge which can be necessary for practical purposes upon this subject may be obtained at anatomical lectures, or by verbal descriptions from anatomical works. But we are convinced that more information may be obtained upon it, and more unfading knowledge, from a few hours' study of really good and correct plates, *of a large size*, than can be derived from any lectures, or any books, without such assistance. The parts are too minute to be seen by the majority of pupils in an anatomical theatre, and a mere verbal description of them cannot convey to the mind half so clear and fixed idea of them as well-executed engravings.

We lay particular stress upon the *size* of the plates, because we deem this a very important point. Small anatomical engravings are necessarily confused; if many parts are illustrated in one drawing, the letters of reference are with great difficulty made out. The magnificent scale upon which Mr. Swan's engravings are executed renders every part he illustrates clear and perceptible at a glance.

We cannot bestow higher, nor can we bestow more just praise, than by saying, that all that the graphic art can achieve for the instruction of the student in one of the most intricate parts of anatomy has been achieved by Mr. Swan, in these beautiful and correct "demonstrations of the nerves of the human body." This is precisely the kind of work which should be added to every scientific library, and all public and private teachers of anatomy will be enabled, by its help, very materially to assist their pupils in obtaining a lasting knowledge of that part of anatomy of which few obtain sufficient knowledge, and which is of such vast importance both in a physiological and pathological point of view.

---

Parts XIV. XV. XVI. *The Principles and Practice of Obstetric Medicine, in a Series of Systematic Dissertations on Midwifery, and on the Diseases of Women and Children. Illustrated by numerous Plates.* By David D. Davies, m.d., m.r.s.l., &c. —4to. London: John Taylor.

In these three parts of this very useful work for students, and even practitioners, there is much practical information, upon the subjects of Amenorrhœa, Menorrhagia, Dysmenorrhœa, Lucorrhœa, &c. In the relation of some of his cases, Dr. Davis is occasionally somewhat too diffuse, and too many ornamental digressions are indulged in. We are by no means averse to an easy and even amusing style of writing, even upon medical topics; it is perhaps the surest attraction for the student, and well adapted to instruct, without wearying him; Dr. Gooch's is a good example of what we mean, but he had always the good taste to confine himself within proper bounds, and to preserve that sobriety of diction which his subject demanded. Dr. Davis sometimes errs on both these points: for, example, it can be of no consequence to the practical reader, nor can the information be at all interesting to him, to be told that a chlorotic young lady falls in love "with a gentleman not more than seven or eight years older than herself;" that he possessed a handsome person, and very elegant manners; "that he had a fine taste for poetry and general literature," and that "the attachment was mutual, and equally ardent upon both sides." As the health of the lady was alone concerned, and we are not told that the gentleman sought for or required the doctor's advice, his embellishments might safely have been left unnumbered and untold. Almost every page presents passages of a similar kind; and, if a student were accidentally to cast his eye upon them, he could never imagine the book was from a serious author upon a serious subject. Such novel-like morsels are utterly out of place.

Dr. Davis's work possesses many and solid claims to the attention of the student, and we should be sorry that those claims should be at all weakened by the fault we have hinted at, and which, in future, may be so easily avoided.

---

Part II., February 1833. *Principles and Illustrations of Morbid Anatomy; adapted to the Elements of* M. Andral, *and to the Cyclopædia of Practical Medicine (with which it will correspond in size;) being a complete Series of coloured Lithographic Drawings, from Originals by the Author. With Descriptions and summary Allusions to Cases, Symptoms, Treatment, &c. Designed to constitute an Appendix to Works on the Practice of Physic, and to facilitate the Study of Morbid Anatomy in connexion with Symptoms.* By J. Hope, m.d., f.r.s., Physician to the St. Mary-le-bone Infirmary, &c.—London; Whittaker.

The diseases illustrated and briefly described in this part of Dr. Hope's useful work, are tubercular consumption, pulmonary apoplexy, emphysema of the lungs, encephaloid tumor of the lungs, melanosis, black pulmonary matter, œdema of the lungs, and ossification of the pulmonary substance, and hydatids. The plates are four in number, and contain together twenty-three figures illustrative of the various morbid appearances presented by the diseases above mentioned: they are much better executed than those contained in the first part of the work; the drawing is correct, and the colouring sober and natural.

---

No. I. *Transactions of the Society for the Encouragement of Arts, Manufactures, and Commerce, for the Session* 1831-32.—Society's House, Adelphi, 1832. 8vo. pp. 222; plates, 10.

Although our *medical*, as is most fitting, greatly preponderates over our more general *physical* department, still we do not disregard the rapid progress which correlative sciences are making, and especially those which influence and may advantage anatomical and physiological pursuits: and, if there is any one point at which we more particularly rejoice, in the universal improvement of our profession, it is the more philosophic character which all ranks deservedly enjoy, from the great attention they have lately paid to the general physical sciences, without in the least neglecting those which are more peculiarly their own. Medical men have done much in all the natural sciences, and we are jealous that they should still march in the van: and this they promise to do, by the more liberal schemes of education which our Halls and Colleges now demand.

Among a vast number of curious and important communications on the sciences and arts, there will be found a very able and interesting paper, describing a new form of *dissecting microscope*, and detailing some microscopic observations, contributed by a medical student to the present volume of Transactions, to which our attention has been directed, and which has given rise to the foregoing observations.

It will be recollected that, in former numbers, we have noticed various improvements in the construction of microscopes, introduced by Messrs. Varley, Valentine, Solly, Pritchard, &c., and have recorded some of the curious discoveries made by their means; and we have now the additional pleasure of stating, that several advances have, within the past year, been made by Messrs. Turrell, Holland, and Slack; detailed accounts of which are published in the Transactions of the Society of Arts now before us.

Messrs. Turrell and Slack's improvements are in the mechanical construction of the instruments, and are much to be commended; but Mr. Holland's new lens, which he calls a "triplet," is by far the most important. Indeed, it is a glass which no one can venture to be without; its defining power being much superior to either Wollaston's or Herschell's doublets.

Mr. Slack's dissecting microscope has likewise our warm approval; its convenience and cheapness must recommend it to every one; and, in proof of its capabilities, the very interesting paper to which we have already referred, is subjoined: from which we should have made some extracts, did not the details require the figures which, in the original, have been supplied by the liberality of Mr. R. H. Solly, a great friend and patron of the arts, and who delights in forwarding and encouraging practical science, and in assisting those who are labouring in the pursuit. We are therefore obliged, although unwillingly, to refer our readers to the original essays, without enriching our pages by a sample; and shall only in conclusion add, that Mr. Holland lives at No. 7, Manor place, Walworth; and that Mr. Slack's dissecting microscope is made by Ross, of St. John's square, Clerkenwell.

---

*British Flowering Plants.* By W. Baxter, Curator of the Oxford Botanic Garden.

*English Botany.* Small Edition. By C. E. Sowerby, F.L.S. &c.

*Medical Botany.* New Edition. By Gilbert T. Burnett.

Two of the above works we have previously announced, and now that they have so far proceeded, and six numbers of each are before the public, we think that we should be wanting in our duty, were we not to state that, as far as they have gone, they have fully performed the promises made in the prospectuses, and cannot fail to be of much service, the two first to the general, and the last (as we hope) to the medical botanist.

Mr. Baxter is well known for his unwearied observations, and the correctness of his judgment; therefore, figures and descriptions coming from a person possessing such extended practical information, will, when completed, form a most valuable work of reference.

The character of Smith and Sowerby's "English Botany" has been too long established to need any commendation now. It is only necessary for us to say, that, to those persons who shrunk from the expense of the large, this small edition must be very acceptable.

Of the new edition of Medical Botany, we had rather let others speak than ourselves: we will only mention that, in the five numbers now published, two figures have been re-engraved, viz. Stramonium and Digitalis, and several of the others have had important additions and alterations made; besides which, the letterpress in every number has been much increased, and in some nearly, if not quite, doubled in extent. To these circumstances it is doubtless that it has received the favorable notice of most of our contemporaries; the opinions of two or three of whom we subjoin, as we cannot here venture to express an opinion of our own.

"This new edition, greatly improved in value and reduced in price, will be found a most acceptable present to the botanical student, and the lover of botany in general."—*Dr. Johnson's Review, January* 1833.

"Three numbers of this work are now before the public, and cannot fail to have made a favorable impression on those who have examined them. Each number contains four coloured plates, not of the kind so often called, which resemble nothing in nature; but well executed, and in some instances, highly finished, engravings. Some of them are really excellent: nothing, for instance, can be better than the representations of the Hyoscyamus Niger, and the Taraxacum. The Belladonna, too, and several others of the twelve before us, are beautifully executed. Each plate is accompanied with a botanical, chemical, and medical, description of the plant, which is clearly and succinctly given, and followed by formulæ for its exhibition. The price is amazingly moderate, 2s. 6d. per number, and the work deserving of every encouragement."—*Med. Gazette, December* 15, 1832.

"We are happy to perceive that the re-publication of this valuable work progresses well; it is greatly improved, enlarged, and published at a less expense than the former edition."—*Medical and Surgical Journal.*

